# Microscopic distribution of alkaloids in freeze-fixed stems of *Phellodendron amurense*

**DOI:** 10.3389/fpls.2023.1203768

**Published:** 2023-06-02

**Authors:** Qinyue Gong, Dan Aoki, Yasuyuki Matsushita, Masato Yoshida, Toru Taniguchi, Keita Endoh, Kazuhiko Fukushima

**Affiliations:** ^1^ Graduate School of Bioagricultural Sciences, Nagoya University, Nagoya, Aichi, Japan; ^2^ Graduate School of Agriculture, Tokyo University of Agriculture and Technology, Fuchu, Tokyo, Japan; ^3^ Forest Bio-Research Center, Forestry and Forest Products Research Institute, Hitachi, Ibaraki, Japan; ^4^ Forest Tree Breeding Center, Forestry and Forest Products Research Institute, Hitachi, Ibaraki, Japan

**Keywords:** *Phellodendron amurense*, Rutaceae, cryo-TOF-SIMS/SEM, mass spectrometry imaging, alkaloids

## Abstract

**Introduction:**

*Phellodendron amurense* Rupr. contains rich alkaloids, which have been extensively applied in clinical treatments for their various biological activities. However, detailed microscopic distribution and roles of such alkaloids in *P. amurense* stem still need to be clarified.

**Methods:**

In this study, the distribution of eight alkaloids in the transverse surface of freeze-fixed *P. amurense* stems in fall and summer has been visualized by cryo-time-of-flight secondary ion mass spectrometry and scanning electron microscopy (cryo-TOF-SIMS/SEM), which was found in living tissues with relative contents of different alkaloids varying with the position. In addition, the contents of these alkaloids quantified by high-performance liquid chromatography (HPLC) analysis suggested the seasonal variation from fall to the following summer.

**Results and discussion:**

Distribution of eight alkaloids in the freeze-fixed stems of *P. amurense* from fall and summer seasons has been visualized and assigned into specific living tissues, with relative contents varying in different positions with seasons, which suggested their possible roles in the physiological processes of the plant itself or plant responding to changes in the surrounding conditions.

**Conclusion:**

This study provided a significant basis for further discussion of the genes or enzymes involved in these processes, which will contribute to investigating biosynthetic pathways and specific *in planta* roles of alkaloids.

## 1 Introduction

Plants produce and store abundant secondary metabolites with significant physiological and ecological functions. During the evolution of plants, the prominent defense role of secondary metabolites towards herbivores and pathogens (bacteria, fungi, and even viruses) has been revealed (Levin, 1976; Swain, 1977). In addition, secondary metabolites were also found to serve as nitrogen storage compounds, UV-protectants, and signal compounds attracting pollinating or seed-dispersing animals in some plant species (Schäfer and Wink, 2009). Among all the secondary metabolites of higher plants, alkaloids form a considerable part of secondary metabolites containing nitrogen (Wink, 2008). Therefore, not only their pharmacological effects have been extensively studied, but the microscopic distribution (Ateacha et al., 2018; He et al., 2022) and *in planta* roles of alkaloids have also received significant attention.

*Phellodendron amurense* Rupr. belongs to the family *Rutaceae* and natively grows in northern China, Korea, and Japan. It was reported that *P. amurense* contains rich alkaloids, including berberine, palmatine, jatrorrhizine, magnoflorine, and phellodendrine (Mori et al., 1994; Xian et al., 2014; Wang et al., 2015), which exhibit various biological activities and have been widely used in clinical treatments for their antifungal (Xiao et al., 2015), antibacterial (Tsujii et al., 2020), anti-inflammatory (Lee et al., 2012; Jung et al., 2017), and possible anticancer effects (Muralimanoharan et al., 2009; James et al., 2011; Hambright et al., 2015; Balážová et al., 2022). However, the detailed microscopic distribution and roles of alkaloids in the *P. amurense* stem remain unclear.

Mass spectrometry imaging has become an effective technique for simultaneously acquiring chemical and positional information on target compounds. A measurement system including a glove box (N_2_ environment, −20°C), cryo-vacuum shuttle, time-of-flight secondary ion mass spectrometer (TOF-SIMS), and scanning electron microscope (SEM) has been developed (Kuroda et al., 2013; Masumi et al., 2014; Aoki et al., 2022). In this system, a fresh surface of the frozen plant sample could be appropriately prepared in the glove box to avoid frosting or sublimation, then transferred by cryo-vacuum shuttle to achieve cryo-TOF-SIMS and cryo-SEM analysis. This system has realized the visualization of salicifoline in freeze-fixed stems of *Magnolia kobus* (Okumura et al., 2017), which proved the possibility of achieving mapping of ionic compounds of a trace amount in frozen and hydrated plant samples. Compared to existing techniques, cryo-TOF-SIMS analysis shows excellent potential for non-destructive *in situ* analysis of water-soluble small-molecule components in plants at high sensitivity.

In this study, the distribution of eight alkaloids in the transverse surface of freeze-fixed *P. amurense* stems from the fall and summer seasons has been visualized by cryo-TOF-SIMS/SEM. The amount of alkaloids was quantified by high-performance liquid chromatography (HPLC) using whole blocks or serial tangential sections from *P. amurense* stem for verification. Through optical microscopic observation, the distribution of alkaloids was further assigned into specific tissues and discussed with their possible *in planta* roles.

## 2 Results

### 2.1 Radial quantitative distributions of berberine and palmatine by HPLC

**Figure 1:**
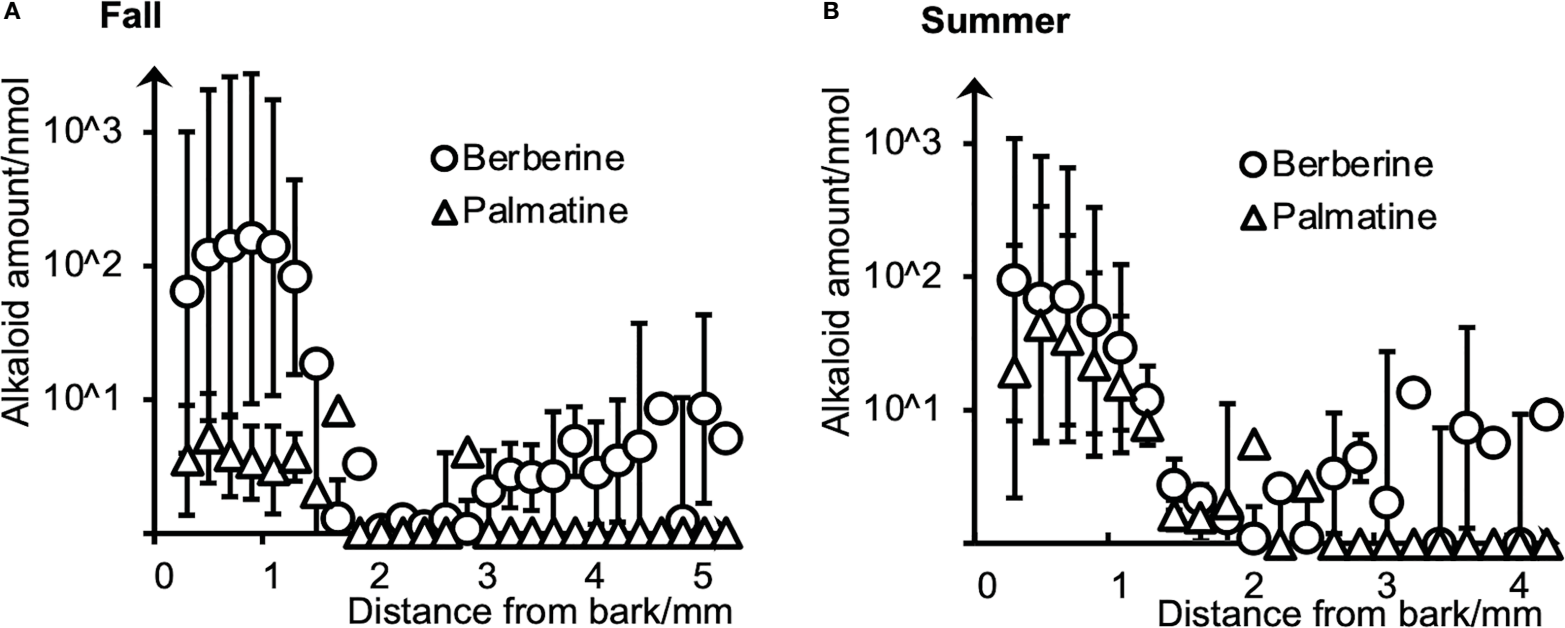
Radial distributions of berberine and palmatine in **(A)** fall and **(B)** summer *P. amurense* stems quantified by HPLC using serial tangential sections. Means and standard errors for each sample were obtained from three measurements (n = 3) using different sample blocks from the same disk.

To evaluate the amounts and radial distributions of berberine and palmatine in *P. amurense*, freeze-fixed blocks from the stems of *P. amurense* sampled in the fall and summer seasons were cut into serial tangential sections of 100-μm thickness. Every two serial sections (as one sample) were extracted with acetonitrile (ACN) and measured by HPLC (**Figure 1**).

Berberine and palmatine exhibited similar radial distributions in the stems of *P. amurense* from summer and fall. They were mainly distributed in the phloem region but also in the cambial zone. In the xylem region, berberine was detected in most sections, while palmatine was only in several sections. Regarding the overall amount of alkaloids, berberine had higher content than palmatine in both seasons, which is consistent with the previous studies (Xian et al., 2014). However, from fall to summer, there was a slight decrease in the content of berberine and a significant increase in the content of palmatine.

### 2.2 Amounts of minor alkaloids in fall and summer *P. amurense* stems by HPLC

It was reported that minor alkaloids, including columbamine, jatrorrhizine, magnoflorine, phellodendrine, 8-oxoepiberberine, and tetrahydropalmatine present in trace amounts in the stem of *P. amurense* (Xian et al., 2014). To quantify these minor alkaloids, freeze-fixed blocks from the stems of *P. amurense* in the fall and summer seasons were extracted with a 95% ACN aqueous solution (aq.). Obtained extracts were measured by HPLC at different conditions (**Table 1**) to achieve optimal separation and quantification of target alkaloids.

**Table 1:** HPLC conditions for quantifying minor alkaloids.

HPLC Condition	Condition 1		Condition 2		Condition 3	
**Quantified Compounds**	Tetrahydropalmatine 8-Oxoepiberberine		Phellodendrine Magnoflorine		Jatrorrhizine Columbamine	
**Buffer A**	0.1% FA		10 mM NH_4_COOH (pH = 3.6)		10 mM NH_4_COOH (pH = 4.8)	
**Buffer B**	ACN (0.1% FA)		ACN		ACN	
**Gradient**	Time/min	% (*v*/*v*) B	Time/min	% (*v*/*v*) B	Time/min	% (*v*/*v*) B
	0	5	0	18	0	18
	5	20	14	44	14	44
	20	30	17	80	17	80
	20.1	95	20	80	20	80
	30	95	21	18	21	18
	30.1	5	30	18	30	18
	35	5				

Five of the minor alkaloids got separated and quantified at three different conditions, while it was hard to quantify tetrahydropalmatine separately: the signal overlapped with that of columbamine in Condition 1 and was not significantly detected in Conditions 2 and 3 (**Figure S1**). In this case, the difference between the total area of the overlapping peak and the equivalent peak area of columbamine, which had been quantified in Condition 3, was regarded as the equal peak area of tetrahydropalmatine in Condition 1 and used for its quantification.

Quantification results of six minor alkaloids by HPLC are shown in **Table 2**. Compared with the contents in fall samples, five alkaloids, including jatrorrhizine, columbamine, magnoflorine, phellodendrine, and 8-oxoepiberberine had a higher content in summer samples, while tetrahydropalmatine was of slightly lower content.

**Table 2:** Contents of minor alkaloids in fall and summer *P. amurense* stems quantified by HPLC.

*c*(alkaloid)/(μmol·g^−1^)	Fall	Summer
Columbamine	0.041	0.205
Jatrorrhizine	0.099	0.569
Magnoflorine	1.410	3.951
Phellodendrine	4.635	8.395
8-Oxoepiberberine	0.040	0.123
Tetrahydropalmatine	0.250	0.229

Means for each sample were obtained from two sets of measurements (n = 2) using sample blocks from two different disks.

### 2.3 Cryo-TOF-SIMS spectra of standard chemicals and freeze-fixed *P. amurense* stems in fall and summer

**Figure 2:**
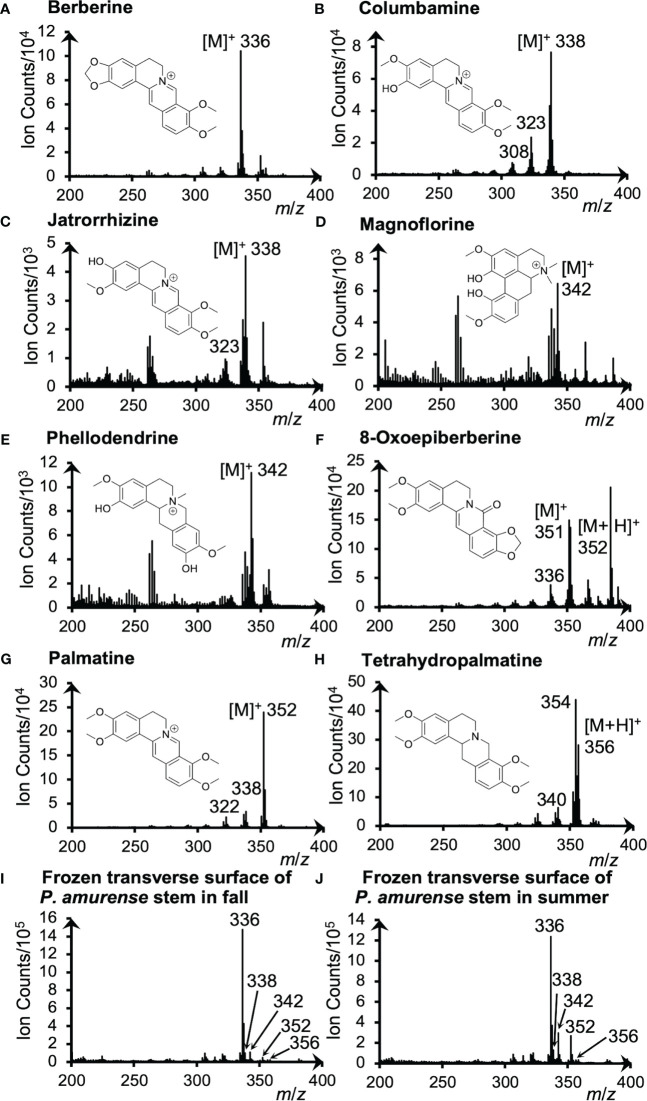
Cryo-TOF-SIMS spectra and chemical structures of **(A)** berberine, **(B)** columbamine, **(C)** jatrorrhizine, **(D)** magnoflorine, **(E)** phellodendrine, **(F)** 8-oxoepiberberine, **(G)** palmatine and **(H)** tetrahydropalmatine. Cryo-TOF-SIMS spectra obtained from the frozen, hydrated transverse surface of a *P. amurense* stem from **(I)** fall and **(J)** summer in the region containing phloem, cambial zone, and xylem. Alkaloid standard chemicals were dissolved at *ca.* 50 mM in ACN and frozen for measurements.

Alkaloid standards were measured by cryo-TOF-SIMS to determine their specific secondary ions. The acquired standard and the typical spectra obtained from the transverse surface of freeze-fixed *P. amurense* stems in fall and summer are shown in **Figure 2**.

For quaternary ammonium alkaloids, including berberine, columbamine, jatrorrhizine, magnoflorine, phellodendrine, and palmatine, the strongest signals were detected at *m*/*z* 336, 338, 342, and 352, respectively, indicating their representative secondary ions to be the molecular ions [M]^+^. However, [M+H]^+^ ion at *m*/*z* 356 was detected as the strongest ion for the tertiary ammonium alkaloid tetrahydropalmatine. As for another tertiary ammonium alkaloid, 8-oxoepiberberine, since [M]^+^ ion at *m*/*z* 351 and [M+H]^+^ ion at *m*/*z* 352 were detected with similar intensities, visualizations of both ions are discussed in 3.4. Fragment ions were also detected as [M−CH_3_]^+^, [M−CH_3_+H]^+^, or [M−CH_3_−CH_3_]^+^ ions in the spectra of alkaloids (**Figure 2**), but their intensity was low. From these results, it was determined to use [M]^+^ and [M+H]^+^ ions to visualize these alkaloids.

Cryo-TOF-SIMS spectra acquired by measuring the surface of actual samples in fall and summer (**Figure 2I, J**) exhibited the signals of characteristic ions of alkaloids as well. For example, berberine, the alkaloid of the highest amount in the stem of *P. amurense*, was detected with high intensity at *m*/*z* 336. Also, overlapping signals of significant ions with the same *m*/*z* produced by alkaloids were detected at *m*/*z* 338 (columbamine and jatrorrhizine), 342 (magnoflorine and phellodendrine), and 352 (8-oxoepiberberine and palmatine), and tetrahydropalmatine was detected at *m*/*z* 356.

### 2.4 The distribution of alkaloids in freeze-fixed *P. amurense* stems from the fall and summer seasons

**Figure 3:**
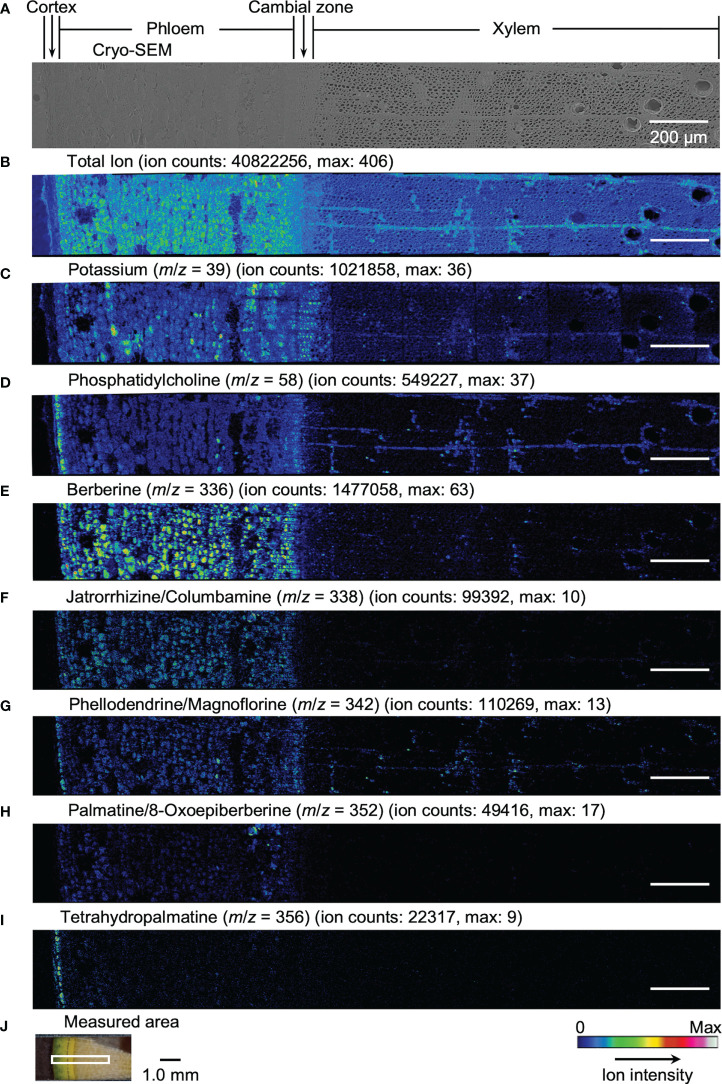
Cryo-TOF-SIMS/SEM images of a freeze-fixed stem of *P. amurense* in fall at the transverse surface. **(A)** Cryo-SEM image of the freeze-etched sample after cryo-TOF-SIMS analysis. Cryo-TOF-SIMS images for positive ions of **(B)** total ions, **(C)** potassium at *m*/*z* 39, **(D)** phosphatidylcholine at *m*/*z* 58, **(E)** berberine at *m*/*z* 336, **(F)** columbamine and jatrorrhizine at *m*/*z* 338, **(G)** magnoflorine and phellodendrine at *m*/*z* 342, **(H)** 8-oxoepiberberine and palmatine at *m*/*z* 352, **(I)** tetrahydropalmatine at *m*/*z* 356. **(J)** An optical microscopy image of a freeze-fixed stem of *P. amurense* from the fall season in the sample holder showing the measured area. Scale bars are 200 μm for **(A-I)** and 1.0 mm for **(J)**.

Results obtained by cryo-TOF-SIMS/SEM analysis of freeze-fixed stems of *P. amurense* in fall and summer are shown in **Figure 3**, **4**. After cryo-TOF-SIMS measurements, the sample blocks were transferred to cryo-SEM for conducting observation (**Figure 3A**, **4A**) of the same measurement area containing phloem, cambial zone and xylem (**Figure 3J**, **4J**) after appropriate freeze-etching (**Figure S2**), which was to enhance the contrast of cryo-SEM images. To further assign detailed tissue structures, sample sections were obtained from *P. amurense* stems in the fall and summer, stained by toluidine blue, and observed by optical microscopy (**Figure 5**).

**Figure 4:**
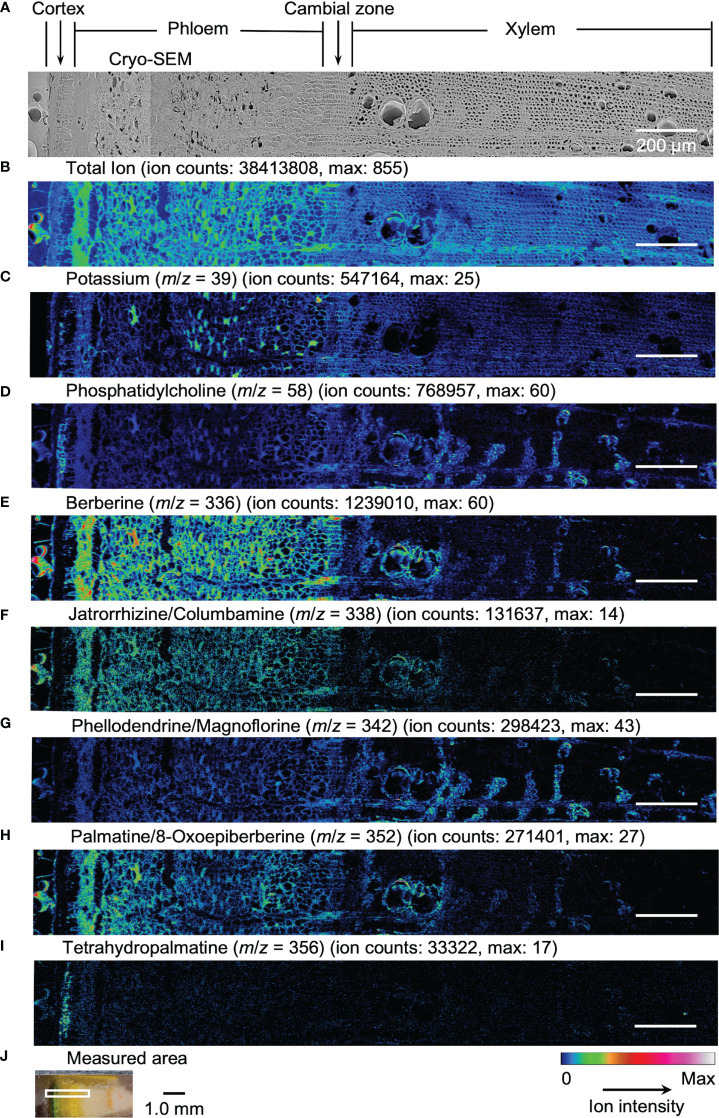
Cryo-TOF-SIMS/SEM images of a freeze-fixed stem of *P. amurense* in summer at the transverse surface. **(A)** Cryo-SEM image of the freeze-etched sample after cryo-TOF-SIMS analysis. Cryo-TOF-SIMS images for positive ions of **(B)** total ions, **(C)** potassium at *m*/*z* 39, **(D)** phosphatidylcholine at *m*/*z* 58, **(E)** berberine at *m*/*z* 336, **(F)** columbamine and jatrorrhizine at *m*/*z* 338, **(G)** magnoflorine and phellodendrine at *m*/*z* 342, **(H)** 8-oxoepiberberine and palmatine at *m*/*z* 352, **(I)** tetrahydropalmatine at *m*/*z* 356. **(J)** Optical microscopy image of a freeze-fixed stem of *P. amurense* from the summer season in the sample holder showing the measured area. Scale bars are 200 μm for (A−I) and 1.0 mm for **(J)**.

**Figure 5:**
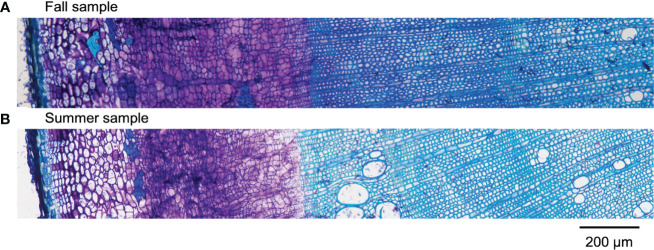
Optical microscopic images of toluidine blue stained sections containing cortex, phloem, cambial zone, and xylem obtained from freeze-fixed stems of *P. amurense* in **(A)** fall and **(B)** summer. The scale bar is 200 μm.

In the results obtained from the transverse surface of the fall *P. amurense* stem, potassium (**Figure 3C**) was specifically detected in the phloem and cambial zone at higher intensity, as well as specific structures in xylem region at a lower intensity, which tended to be similar to the localization of living cells. However, in the results of summer samples, potassium ions were found to have wide distribution over the area measured, except for some specific positions in the phloem (**Figure 4C**). Therefore, the specificity of potassium distribution in the summer sample was not sufficient to represent the biological activity of cells.

Phosphatidylcholine is a major component of plant biological membranes and is considered a good marker for the biological activity of plant cells for its specified detection in living cells. It has been reported that phosphocholine ion ([C_5_H_15_NO_4_P]^+^) of *m*/*z* 184 detected in TOF-SIMS could perform as the marker for phosphatidylcholine ion mapping (Okumura et al., 2017). In addition, the ion of *m*/*z* 58 ([C_3_H_8_N]^+^) was identified as the characteristic and stronger secondary ion derived from phosphocholine in TOF-SIMS analysis (Fletcher et al., 2007; Gunnarsson et al., 2010) (**Figure S3**), by which ray tissues were much more precisely visualized than that by the potassium distribution. From the above points, cryo-TOF-SIMS images of the ion at *m*/*z* 58 (**Figure 3D**, **4D**) are supposed to represent the distribution of the living cells compared to those of *m*/*z* 39 and 184 ions.

Since characteristic ions of jatrorrhizine and columbamine, as well as phellodendrine and magnoflorine, have the same values in *m*/*z*, which resulted in signal overlap in cryo-TOF-SIMS analysis, here we discuss them two-by-two as a whole. [M]^+^ ion of 8-oxoepiberberine with the characteristic ion at *m*/*z* 351 showed the same distribution in cryo-TOF-SIMS images as that of the ion at *m*/*z* 352 (**Figure S4**). Since the signal at *m*/*z* 352 was overlapped by [M]^+^ ion of palmatine and [M+H]^+^ ion of 8-oxoepiberberine, we make an overall discussion on palmatine and 8-oxoepiberberine as well. Eight alkaloids were present in the phloem, cambial zone, and xylem, and the distribution of different alkaloids varied in different tissues. For berberine of *m*/*z* 336 (**Figure 3E**, **4E**), jatrorrhizine and columbamine of *m*/*z* 338 (**Figure 3F**, **4F**), palmatine and 8-oxoepiberberine of *m*/*z* 352 (**Figure 3H**, **4H**), similar distributions of alkaloids are shown for samples from both seasons. They were detected at high intensity in phloem parenchyma cells and the cambial zone. Also, they were found at lower content in the xylem region, with decreasing order from vessel-neighboring parenchyma cells, axial parenchyma cells, to ray cells. They were not detected in the outer cortex or other dead tissues, such as phloem fibers. In contrast, phellodendrine and magnoflorine of *m*/*z* 342 (**Figure 3G**, **4G**) were detected with decreasing order from vessel-neighboring parenchyma and axial parenchyma cells in xylem, ray cells to phloem parenchyma cells and cambial zone, for samples from both seasons. The distribution of phellodendrine and magnoflorine was also found in the inner cortex of the fall sample but not in the summer sample. As for tetrahydropalmatine of *m*/*z* 356 (**Figure 3I**, **4I**), it mainly localized in the inner cortex but was not detected with enough intensity from phloem to xylem in both fall and summer samples.

## 3 Discussion

Accumulation of alkaloids in the inner cortex and phloem should be related to their defensive roles (Hadacek et al., 2010), antimicrobial activities (Schmeller et al., 1997), and antifungal effects (Ma et al., 2000) in plants. Also, activities occurring at low concentration levels, such as biosynthesis as well as inter-cell and inter-tissue transportation, are possible for these pharmacological compounds. Furthermore, it has been reported that alkaloids leached into the soil impact the neighboring plants (Gressel and Holm, 1964; Mccalla and Haskins, 1964). Therefore, it is reasonable that such possible activities that alkaloids carried out in living cells are related to some specific usages in the stem of *P. amurense*. However, as these results were limited to alkaloids of the amount above the limit of detection, and distribution in the axial direction of *P. amurense* stem was not clarified in this study, the detailed mechanism involved needs further discussion to realize an understanding of whole behaviors of alkaloids *in planta*.

From the perspective of seasonal variation in content, berberine and tetrahydropalmatine slightly decreased from fall to the following summer. In contrast, other alkaloids exhibited higher content in summer, among which palmatine considerably increased from fall to summer. The changes in content may be due to the conversion between alkaloids and other nitrogenous compounds in plants. These activities should result from plant growth and physiological changes in different seasons. Putative alkaloid biosynthetic pathways in *Coptis* species involving berberine, jatrorrhizine, palmatine, etc. have been reported (He et al., 2018), so we can speculate by the further study that biosynthetic pathways of alkaloids found in *P. amurense* are possibly correlated as well.

## 4 Materials and methods

### 4.1 Plant materials

Sampling was achieved on 5 November 2019 in the fall and 22 July 2020 in the summer. Each sample disk (thickness of 10 mm) was obtained from *P. amurense* trees (2.0 m in height, 3-year-old) grown in the Botanical Garden at Nagoya University, Higashiyama campus (Nagoya, Japan) and cut into small blocks (circular sector with a radius of *ca.* 4.5 mm and central angle of π/16) containing phloem, cambial zone, and xylem. The blocks were quick-frozen with liquid Freon^®^ 22 (DuPont) at −160°C and stored at −80°C. Detailed procedures are schematically illustrated in **Figure S5**.

### 4.2 Chemicals and reagents

Alkaloid standards including berberine chloride, palmatine chloride, and magnoflorine (SIGMA-Aldrich, MA, USA), tetrahydropalmatine and jatrorrhizine chloride (Tokyo Chemical Industry Co., Ltd., Tokyo, Japan), phellodendrine (Nacalai Tesque Inc., Kyoto, Japan), 8-oxoepiberberine and columbamine (MedChem Express, NJ, USA) were purchased and used as received. Ammonium formate and formic acid (FA) were purchased from Kishida Chemical Co., Ltd. (Osaka, Japan). ACN and distilled water of HPLC grade were purchased from Kanto Chemical Co., Inc. (Tokyo, Japan).

### 4.3 Chromatography measurements for berberine and palmatine

Frozen sample blocks were cut from bark to xylem into serial tangential sections of 100-µm thickness. Every two serial sections were collected in the same plastic tube (1.5 mL in volume) and extracted with 1 mL ACN at 60°C for one hour. HPLC measurements for berberine and palmatine were achieved with Agilent 1100 series LC system (Agilent Technologies Inc.) equipped with a TSKgel ODS-100S column (4.6 mm i.d. × 25 cm, C_18_, 5.0 μm, Tosoh Corp., Japan). Berberine and palmatine were separated by a binary buffer system of 0.1% (*v*/*v*) FA (buffer A) and 0.1% (*v*/*v*) FA in ACN (buffer B) at a flow rate of 1 mL/min. The gradient was 35 min in total and set as follows: from 5 to 20% (*v*/*v*) buffer B in 5 min, from 20 to 30% (*v*/*v*) buffer B in 15 min, from 30 to 95% (*v*/*v*) buffer B in 0.1 min, holding at 95% (*v*/*v*) buffer B for 9.9 min, declining to 5% (*v*/*v*) buffer B in 0.1 min, and holding at 5% (*v*/*v*) buffer B for 4.9 min. All the chromatograms were taken at column temperature 30°C and UV detection wavelength 265 nm. The measurements were carried out in three replicates using three blocks obtained from the same sample disk to evaluate the average amount and standard errors of berberine and palmatine.

### 4.4 Separation and chromatography measurements for the minor alkaloid group

Frozen sample blocks from fall and summer were extracted with 95% ACN aq. (1 mL for every two blocks) at 60°C for one hour. A flash chromatography system (Pure C-810, Büchi, Switzerland) was used to separate the minor alkaloid group from berberine and palmatine using a Flashpure Select C_18_ 4g column (particle diameter 20–35 μm, Büchi). Minor alkaloids were separated by a binary buffer system of 0.1% (*v*/*v*) FA aq. (buffer A) and 0.1% (*v*/*v*) FA in ACN (buffer B) at a flow rate of 15 mL/min. The gradient was 60.7 min in total and set as follows: holding at 5% (*v*/*v*) buffer B in 0.5 min, from 5 to 20% (*v*/*v*) buffer B in 5 min, from 20 to 40% (*v*/*v*) buffer B in 30 min, from 40 to 95% (*v*/*v*) buffer B in 0.1 min, holding at 95% (*v*/*v*) buffer B for 13 min, declining to 5% (*v*/*v*) buffer B in 0.1 min, and holding at 5% (*v*/*v*) buffer B for 12 min. Fractions from 0 to 33 min were collected and concentrated, then re-dissolved with 95% ACN aq. for HPLC analysis.

HPLC quantification of minor alkaloids was achieved with the same LC system equipped with an XSelect CSH C_18_ Column (4.6 mm i.d. × 25 cm, C18, 5.0 μm, Waters Corp., USA). Three conditions (**Table 1**) were applied to achieve better compound separation. All the chromatograms were taken at column temperature 30°C and UV detection wavelength 265 nm. The measurements were performed in two replicates with blocks from two different sample disks to evaluate the average amount of minor alkaloids. MS1 and MS2 spectra (**Figure S6**) of HPLC peaks were obtained for alkaloid standards and the plant samples using Esquire 3000 (Bruker Corp., USA) connected to Agilent 1100 series LC system to confirm the HPLC peak assignments.

### 4.5 Cryo-TOF-SIMS/SEM analyses

Details of the manufactured cryo-TOF-SIMS/SEM system were described previously (Kuroda et al., 2013; Masumi et al., 2014). For each sample from fall and summer, a frozen sample block was fixed in a copper holder by ice embedding and cut in the glove box under a dry N_2_ environment (sample temperature < −30°C) to achieve a clean and flat surface, then transferred to the cryo-TOF-SIMS system by cryo-vacuum shuttle for analysis. Positive ion images were obtained by cryo-TOF-SIMS (TRIFT-III spectrometer, ULVAC-PHI Inc., Japan). 22 keV $\text{A}{\text{u}}_{1}^{+}$ at the current of 5−7 nA was used as the primary ion beam, and a low-energy pulsed electron gun (30.0 eV) was used for surface charge compensation. Other conditions were set as follows: raster size at 300 × 300 μm, measurement time of 10 min, pulse width at 13 ns (non-bunched, image) or 1.8 ns (bunched, spectrum), spot size at 1.0 μm (image), the temperature at −120 to −130°C, vacuum level below 1.0 × 10^−7^ Pa. Standard chemicals of alkaloids were dissolved at *ca.* 50 mM in ACN, dropped on the achieved smooth and flat surface of ice tables (made up of 50 mM KCl solution), and dried up, then measured by cryo-TOF-SIMS in the same procedure in bunched mode.

After cryo-TOF-SIMS measurements, the plant sample block was transferred to cryo-SEM by a cryo-vacuum shuttle. To enhance the contrast of SEM images, the frozen and hydrated sample surface was freeze-etched at −90°C, then observed at around −130°C to obtain images of the same region measured by cryo-TOF-SIMS. The acceleration voltage was set at 1.5 kV, and the working distance was 10 mm.

Obtained cryo-TOF-SIMS images were connected using WinCadence 5.1.2.8 (ULVAC-PHI Inc., Japan) and MATLAB R2019b (The MathWorks Inc., USA) with PLS Toolbox 8.8.1 (Eigenvector Research Inc., USA) without any ion count normalization. Color scales of images were adjusted using ImageJ software (The National Institutes of Health, USA) (Schneider et al., 2012). Cryo-SEM images were connected using Photoshop CS5 Extended (Adobe Systems Inc., USA).

### 4.6 Microscopic observations

Cryo-sections were prepared by Kawamoto’s film method (Kawamoto, 2003; Kawamoto et al., 2021) with some modifications at the submerging steps to obtain better sections (**Figure S7**). First, frozen blocks of *P. amurense* stems in fall and summer were immersed in the embedding medium (SCEM, SECTION-LAB Co., Ltd., Japan) at room temperature for 30 min. Then, thawed blocks were embedded in SCEM and cut into sections at 2-μm thickness using a sliding microtome (REM-710, Yamato Kohki Industrial Co., Ltd., Japan). Before staining, sections obtained were pretreated by the following steps: thawed in the air for 20 s, submerged three times in ethanol for 20 s, 10 s, and 1 min, submerged in acetone for 1 min, submerged in 4% paraformaldehyde solution for 5 min, rinsed under running water for 5 min. After rinsing, sections of 2 μm from the fall block and 4 μm from the summer block were stained with toluidine blue and observed using an optical microscope (BX-60, Olympus Corp., Japan).

## 5 Conclusion

In this study, eight alkaloids, including berberine, columbamine, jatrorrhizine, magnoflorine, phellodendrine, palmatine, 8-oxoepiberberine, and tetrahydropalmatine have been visualized in the transverse surface of freeze-fixed *P. amurense* stems from fall and summer seasons by cryo-TOF-SIMS/SEM. The distribution of alkaloids was found in living tissues, but the relative contents of alkaloids varied at different positions. Such diversified distribution of alkaloids in different positions plays a role in the physiological processes of the plant itself or the plant responding to changes in the surrounding conditions. The present study has brought possibilities for further discussion on genes or enzymes involved in these processes, which would contribute to investigating biosynthetic pathways and the specific *in planta* roles of alkaloids.

## Data availability statement

The raw data supporting the conclusions of this article will be made available by the authors, without undue reservation.

## Author contributions

QG, DA, YM, and KF designed the research. QG, DA, TT, and KE collected the *P. amurense* samples. QG conducted the HPLC experiments. QG and DA performed the cryo-TOF-SIMS/SEM analysis. QG, DA, and MY achieved the microscopic observation and tissue assignment. All the authors discussed the results. QG and DA wrote the manuscript. All authors contributed to the article and approved the submitted version.
