# Supplementary material for: Microscopic distribution of alkaloids in freeze-fixed stems of Phellodendron amurense
Source: Front Plant Sci. 2023 Jun 2;14:1203768. doi: 10.3389/fpls.2023.1203768 (PMC10283038; doi:10.3389/fpls.2023.1203768)
Supplement: Supplementary file 1 [file DataSheet_1.docx]

Supplementary Material

Microscopic Distribution of Alkaloids in Freeze-fixed Stems of *Phellodendron amurense*

Qinyue Gong^1^, Dan Aoki^1^*, Yasuyuki Matsushita^1,2^, Masato Yoshida^1^, Toru Taniguchi^3^, Keita Endoh^4^, Kazuhiko Fukushima^1^

^1^Graduate School of Bioagricultural Sciences, Nagoya University, Nagoya, Japan

^2^Graduate School of Agriculture, Tokyo University of Agriculture and Technology, Fuchu, Japan

^3^Forest Bio-Research Center, Forestry and Forest Products Research Institute, Hitachi, Japan

^4^Forest Tree Breeding Center, Forestry and Forest Products Research Institute, Hitachi, Japan

*** Correspondence:** Corresponding Author: Dan Aoki (aoki.dan@nagoya-u.jp)

Keywords:

*Phellodendron amurense*, *Rutaceae*, cryo-TOF-SIMS/SEM, mass spectrometry imaging, alkaloids

# Supplementary Figures

**Figure S1.** UV chromatograms of minor alkaloid standards at three HPLC conditions showing peak separation, respectively.

**Figure S2.** Cryo-SEM images of freeze-fixed stems of *P. amurense* at transverse surface, obtained at the same positions before and after freeze-etching.

**Figure S3.** Cryo-TOF-SIMS standard spectrum of phosphatidylcholine.

**Figure S4.** Cryo-TOF-SIMS images for positive ions of 8-oxoepiberberine at *m*/*z* 351, and for positive ions of 8-oxoepiberberine and palmatine at *m*/*z* 352, in freeze-fixed stems of *P. amurense* from fall and summer seasons.

**Figure S5.** Experiment Scheme.

**Figure S6.** MS spectra by LC-MS/MS for *P. amurense* sample and standard solutions of eight alkaloids under the corresponding quantitative LC conditions.

**Figure S7.** Modified procedure of section preparation following Kawamoto’s film method.

**Figure S1.** UV chromatograms of minor alkaloid standards at three HPLC conditions showing peak separation, respectively. (A) Peak of 8-oxoepiberberine got separated with the overlapping peak of tetrahydropalmatine and columbamine in Condition 1. (B) Peaks of magnoflorine and phellodendrine got separated in Condition 2. (C) Peaks of columbamine and jatrorrhizine got separated in Condition 3. Standard samples were of concentration at 0.25 g/L.

**Figure S2.** Cryo-SEM images of freeze-fixed stems of *P. amurense* at transverse surface, obtained at the same positions before and after freeze-etching. (A) Phloem, (B) cambial zone and xylem of the fall sample before freeze-etching. (C) Phloem, (D) cambial zone and xylem of the fall sample after freeze-etching. (E) Phloem, (F) cambial zone and xylem of the summer sample before freeze-etching. (G) Phloem, (H) cambial zone and xylem of the summer sample after freeze-etching. Scale bar is 100 μm.

**Figure S3.** Cryo-TOF-SIMS standard spectrum of phosphatidylcholine. Standard chemical was dissolved at 100 mM in 100 mM KCl solution and frozen for the measurement conducted in bunched mode.

**Figure S4.** Cryo-TOF-SIMS images for positive ions of 8-oxoepiberberine at *m*/*z* 351 in freeze-fixed stems of *P. amurense* from (A) fall and (C) summer seasons (binary images), and for positive ions of 8-oxoepiberberine and palmatine at *m*/*z* 352 in freeze-fixed stems of *P. amurense* from (B) fall and (D) summer seasons. Optical microscopy images of freeze-fixed stems of *P. amurense* from (E) fall and (F) summer seasons in the sample holder showing the measured areas. Color bars for each figure indicate the ion intensities from zero to maximum. Scale bars are 200 μm for (A−D) and 1.0 mm for (E−F).


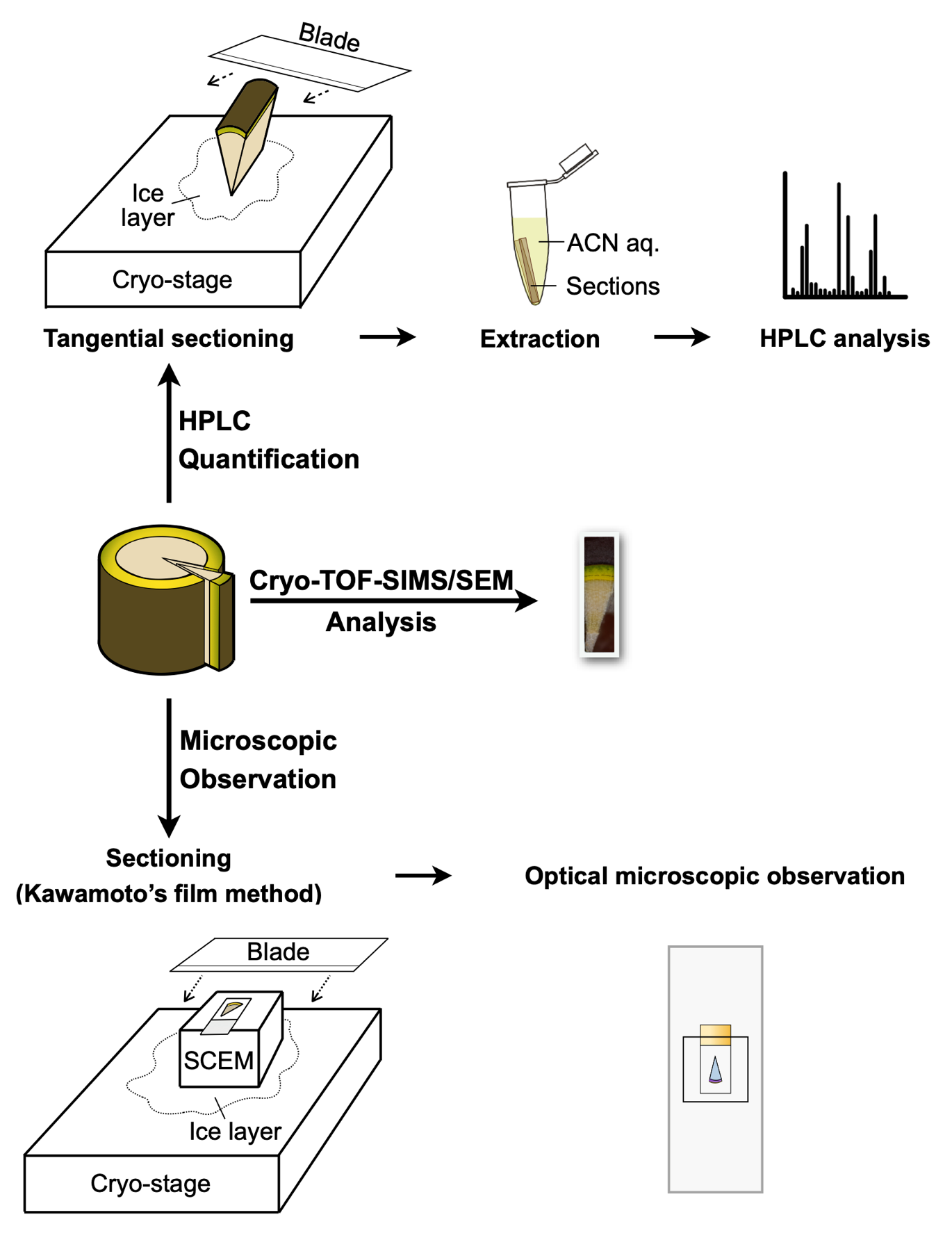


**Figure S5.** Experiment scheme.

**Figure S6.** MS spectra by LC-MS/MS for *P. amurense* sample and standard solutions of eight alkaloids under the corresponding quantitative LC conditions.

(**Figure S6.** Continued)

(**Figure S6.** Continued)

(**Figure S6.** Continued)

**Figure S7.** Modified procedure of section preparation following Kawamoto’s film method.
